# Understanding Online Health Groups for Depression: Social Network and Linguistic Perspectives

**DOI:** 10.2196/jmir.5042

**Published:** 2016-03-10

**Authors:** Ronghua Xu, Qingpeng Zhang

**Affiliations:** ^1^ Department of Systems Engineering and Engineering Management City University of Hong Kong Kowloon China (Hong Kong); ^2^ Shenzhen Research Institute of City University of Hong Kong Shenzhen China

**Keywords:** mental health, depression, social media, information science, online health group, social network analysis

## Abstract

**Background:**

Mental health problems have become increasingly prevalent in the past decade. With the advance of Web 2.0 technologies, social media present a novel platform for Web users to form online health groups. Members of online health groups discuss health-related issues and mutually help one another by anonymously revealing their mental conditions, sharing personal experiences, exchanging health information, and providing suggestions and support. The conversations in online health groups contain valuable information to facilitate the understanding of their mutual help behaviors and their mental health problems.

**Objective:**

We aimed to characterize the conversations in a major online health group for major depressive disorder (MDD) patients in a popular Chinese social media platform. In particular, we intended to explain how Web users discuss depression-related issues from the perspective of the social networks and linguistic patterns revealed by the members’ conversations.

**Methods:**

Social network analysis and linguistic analysis were employed to characterize the social structure and linguistic patterns, respectively. Furthermore, we integrated both perspectives to exploit the hidden relations between them.

**Results:**

We found an intensive use of self-focus words and negative affect words. In general, group members used a higher proportion of negative affect words than positive affect words. The social network of the MDD group for depression possessed small-world and scale-free properties, with a much higher reciprocity ratio and clustering coefficient value as compared to the networks of other social media platforms and classic network models. We observed a number of interesting relationships, either strong correlations or convergent trends, between the topological properties and linguistic properties of the MDD group members.

**Conclusions:**

(1) The MDD group members have the characteristics of self-preoccupation and negative thought content, according to Beck’s cognitive theory of depression; (2) the social structure of the MDD group is much stickier than those of other social media groups, indicating the tendency of mutual communications and efficient spread of information in the MDD group; and (3) the linguistic patterns of MDD members are associated with their topological positions in the social network.

##  Introduction

Mental health problems, such as anxiety, bipolar disorder, and depression, have become increasingly prevalent in recent years. According to a recent report from the World Health Organization [1], one in four people in the world will be affected by mental disorders at some point in their lives. Among the various problems, depression is prevalent and could lead to other mental disorders [2]. More than 350 million people suffer from depression worldwide. Once being depressed, it is common to recur and will often last for many years [3]. However, due to the stigma and discrimination, depression is widely unreported and many symptoms are unrecognized [4]. The situation is even worse in China because of the rapid pace and high pressure of society, and the Chinese *Mianzi* (meaning “face” or a person's own sense of dignity or prestige) culture.

During the past decade, social media has played an increasingly important role in the promotion of mental health. It has been widely utilized by people to deal with health-related issues because of its publicity, broad reach, usability, and immediacy [5]. People use social media to acquire health information and seek social awareness [6]. In addition, they also form online health groups to grant and receive health suggestions and social support [7-9]. With respect to mental disorders, various online groups (either predefined by the platform or created by the users) encourage patients to anonymously share their innermost feelings and talk about their experiences and problems, which may not be possible in real life [10]. Therefore, the wide adoption of social media platforms presents an ideal data source and a testbed for researchers to study mental health problems from a brand new perspective [6,7,10-13].

Many research works have used social media for the detection and monitoring of depression. In Ramirez-Esparza et al [11], the authors performed content analysis of online forums about mental health topics in both English and Spanish. They found that linguistic differences existed between depressed and nondepressed posts, indicating that depression symptoms were revealed by the content in online media. Park et al [12] compared the tweets of people without mental disorders and those of people diagnosed by psychological tests with depression and showed that social media contained useful signals of depression, such as emotion words and language use styles. Similar studies with the Facebook data of college students verified that the symptoms of depression were consistent both online and offline [13,14]. Although the patterns of language use were effective in the detection of depression, there is little understanding of Web users’ conversations, which contain important information on social interactions as well as language use styles [15].

In addition to linguistic patterns, the topological properties of the social networks formed in social media also play an important role in the understanding of depression-related issues [16,17]. Social networks not only represent the communication among social media users, but also implicate the social structure of the whole group [10,18,19]. More importantly, social condition, which is one of the major causes and manifestations of mental health problems, could be derived from the analysis of social networks [20,21]. However, previous research works either focused on the linguistic factors or the social network factors with respect to mental problems; few have focused on both factors. This work attempts to study depression by exploring both the social structure and language use.

In this paper, we investigate online health groups for depression with data from Douban*,* a popular social media platform in China. Douban allows users to create interest groups so that users with similar interests can get together to discuss related topics, such as a specific disease, a city, a hobby (eg, photography), etc. Among various interest groups, more than 1000 are related to mental health, with more than 1 million members. We named these health-related interest groups as “online health groups” and chose the most popular Douban group related to depression, called the major depressive disorder (MDD) group [22]. We selected this group because it is the largest active Chinese online health group specifically designed for people who have been clinically diagnosed with depression [23]. We use “MDD group” to refer to this online health group in this paper.

In this research, we attempt to answer the following questions:

1. What are the unique language use patterns in the conversations of the MDD group?
2. What are the unique characteristics of the social networks formed by the conversations in the MDD group?
3. What are the relations between the language use patterns and the topological properties of members in the MDD group?

## Methods

### Data Collection

The group we studied was the MDD group on Douban [22] compared to minor and subsyndromal depression. The MDD group is specifically designed for people who have been clinically diagnosed with depression as compared to other online health groups for people with depressive moods. To provide mutual help, the group encourages its members to discuss their problems and exchange possible therapies and suggestions to cope with negative emotions and tough experiences with the disorder. The MDD group was founded on August 26, 2008; 5050 members joined the discussion since then. According to the purpose and scope of the group, all members have self-tested or have been clinically diagnosed with depression. They grouped together to give experience, understanding, support, and help to one another during the course of the illness.

In Douban interest groups, members use an anonymous user ID to communicate with one another. Once the group is created, any Douban user can start a discussion thread in this group. Each thread contains the title, the user ID of the creator (initiator), the created time, and the content of the message posted by the creator. MDD group members can join the discussion by posting messages in a certain thread. A message contains the user ID of the member, the post time, and the content of the message. A member can specify the message is a reply to the original post of the initiator or to an existing message posted by another member, thus forming a reply-to relationship.

**Figure 1:**
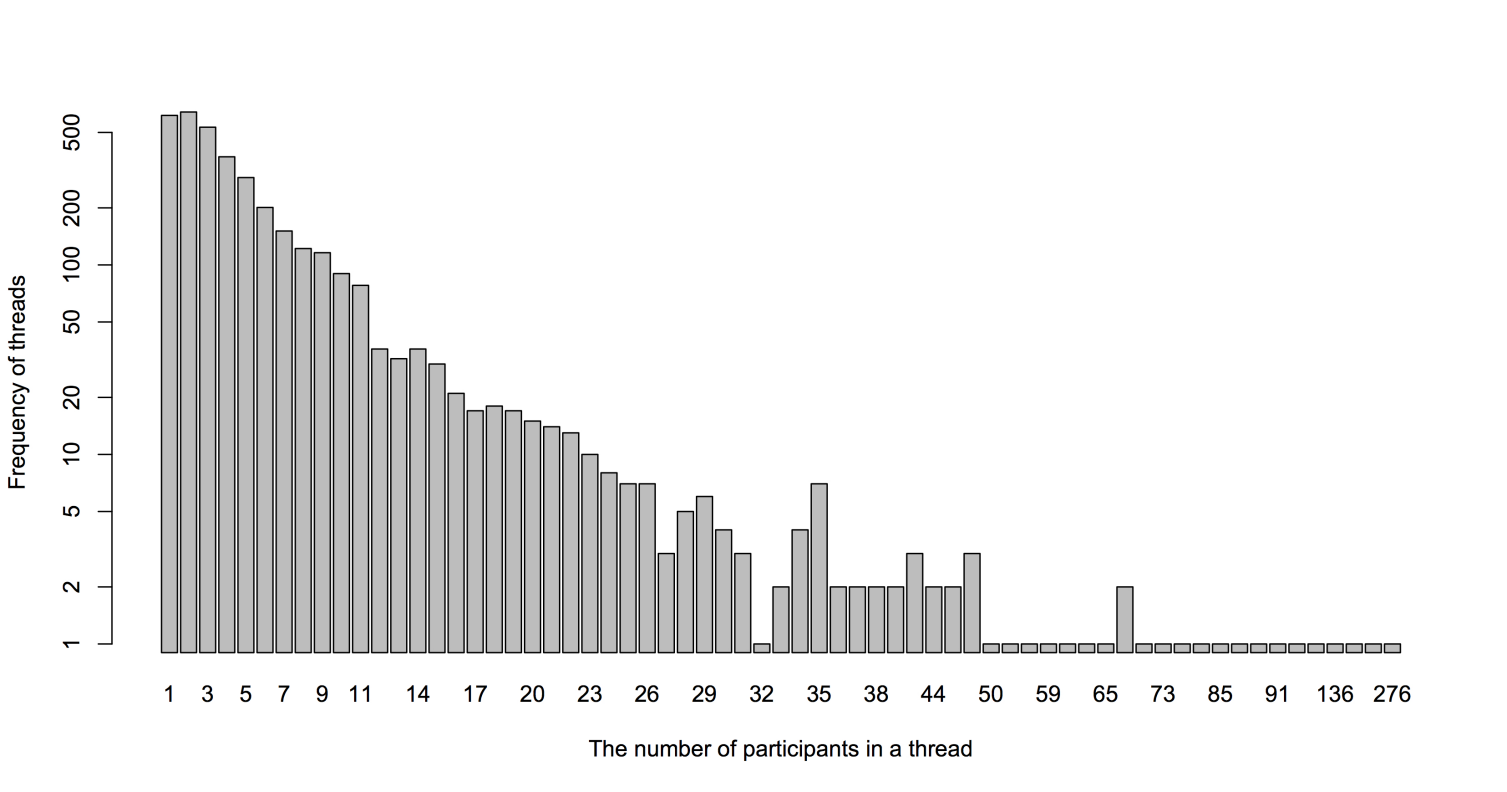
Distribution of the number of participants in a thread.

We focused on the textual content of messages and the reply-to relationship between members in the MDD group. The messages conveyed valuable information about the linguistic patterns of the members. The reply-to relationship among the members represented their mutual conversations in the MDD group. We collected the full information of 3700 threads, 40,357 messages, and from 5050 members from the founding date on August 26, 2008 to January 6, 2015. Figure 1 shows the distribution of the number of participants in a thread in log-scale. In general, the frequency peaks at two participants and then diminishes along with an increase in the number of participants. The largest number of participants in a thread was 276, indicating that there were 276 unique user IDs participating in the discussion in the corresponding thread.

### Text Processing

To process Chinese content, we employed a popular open-sourced toolkit, jieba, to perform the Chinese segmentation task [24]. Then the segmented words were fed into Linguistic Inquiry Word Count (LIWC), a validated and well-adopted toolkit for psychometric analysis in mental expression research [25,26]. LIWC can reveal the language use associated with a psychological disorder by classifying the input words into linguistic categories (eg, articles, numbers, and pronouns), psychological categories (eg, cognitive, affective, and social), or personal concern categories (eg, death, home, and school). The detailed explanations of the categories can be found on the LIWC website [27]. The resulting categories are standardized to be the occurrence rate of corresponding categorical words in the messages.

To analyze Chinese text, we used the Chinese version dictionary of Linguistic Inquiry Word Count (SC-LIWC) [28]. The dictionary contains 7444 Chinese words from 64 subcategories out of seven higher-level main categories. The seven main categories are function words (eg, 或许 [maybe], 许多 [many], 那些 [those]), social processes (eg, 家人 [husband], 接纳 [accept], 打招呼 [buddy]), affective processes (eg, 气愤 [annoyed], 感恩 [grief], 失望 [sad]), cognitive processes (eg, 理解 [know], 选择 [select], 质疑 [guess]), perceptual processes (eg, 温暖 [feeling], 经验 [heard], 注视 [see]), biological processes (eg, 头晕 [dizzy], 拥抱 [hug], 流汗 [sweat]), and relativity (eg, 以前 [before], 相比 [compare], 达到 [achieve]) [28]. Based on the dictionary, the LIWC toolkit was able to extract 75 dimensions of language uses, including 64 subcategories and seven main categories, and four descriptive properties: the word count, the tag rate, the number of words per sentence, and the number of words longer than six letters. These linguistic properties could be further used as proxies of depressive and suicidal signs relevant to depression and other mental disorders. Because we were investigating the MDD group, we selected a set of properties closely related to depression, including word count, the seven main categories, and the five subcategories related to emotion and pronouns.

### Network Construction and Analysis Methods

We constructed the social network formed by the conversations (indicated by the reply-to relationship of messages) of the members. A unique node in the network represented a unique user ID. An edge between two nodes represented the existence of a reply-to relationship between the two corresponding nodes. The direction of an edge is from the user who posted replies to the user who posted the original message. There could be multiple edges between two nodes because there could be multiple replies. The procedure of network construction was as follows:

1. When a Douban user joined the MDD group, either by initiating a new discussion thread or posting a message in an existing discussion thread, a new node representing the new member was generated and a new edge representing his or her behavior was constructed based on the following rules.
2. When a user *A* initiated a new discussion thread, a self-loop of *A* was added to denote the thread initiation.
3. When a user *B* posted a message in a discussion thread in the group, a new directed edge from *B* to the initiator of the thread was constructed.
4. When a user *C* posted a message in a discussion thread and the message was a reply to another message posted by a user *D*, a directed edge from *C* to *D* was constructed. If *C* posted a reply to a message posted by *C* (*D* was *C*), a self-loop of *C* was formed.

We named the constructed network the MDD network, which was a directed network with self-loops and multiple edges.

We adopted a set of well-established network metrics to analyze the MDD network, including degree centrality (including both in-degree and out-degree), average shortest path length, betweenness centrality, and clustering coefficient. For detailed explanations of these metrics, please refer to Newman [16]. In the MDD network, the in-degree of a node represents the number of replying messages that the corresponding user received from other users. The out-degree of a node represents the number of messages that the corresponding user posted as a reply to another user. The average shortest path length of a node is the mean of the shortest path lengths to all other nodes that could be reached through a directed path in the network. The betweenness centrality of a node is the proportion of the shortest paths between any pairs of two nodes traversing through this node. It represents the importance of this node for the interactions of other nodes in the network. The clustering coefficient of a node is defined as the proportion of the existing edges among the neighbors of this node over all the possible edges among his or her neighbors. It measures the connectivity, transitivity, and clustering intensity among users in the MDD group. From the view of the whole network, we also analyzed the network density, network diameter, and network reciprocity. The network density is defined by the ratio of the number of existing edges over the number of all possible edges. The network diameter is the largest value of the average shortest path length of all pairs of nodes. The network reciprocity is the tendency of node pairs to form mutual reply-to edges between one another.

In addition, the Bow-Tie model was used to examine the general structure of the MDD network in more detail. In the Bow-Tie model [29,30], the central core of the group is represented by the largest strongly connected component (SCC), within which any user can reach any other users through a directed path. The IN component consists of users who only replied to the members in SCC, and did not receive any response from SCC users. The OUT component consists of the users who only received replies from the users in SCC, but did not post any replies to SSC users. TENDRILS component consists of users who connected to either IN or OUT. TUBES component consists of non-SCC users who connected two users from IN and OUT components. The rest nodes are in DISC, indicating that they are disconnected components.

To have an in-depth understanding of the formation of the MDD group, we compared the network properties of the MDD network with other online health groups, social media communities, and the Web. We also employed the classic Erdös-Rényi random network model [31] and Barabási-Albert preferential attachment model [32] to simulate the MDD network. To generate networks with the same number of nodes and edges as the MDD network, we generated a Barabási-Albert model by starting with a complete network of five nodes and adding a new node and seven edges at each step. In addition, we also abstracted the undirected network, called MDD-friend network, by excluding the direction of edges in the MDD network. We assumed that if two members have exchanged messages, they possessed a weak social relation. In this way, we can also compared the MDD-friend network with undirected networks formed in other online health groups, particularly the network of MedHelp presented in Vydiswaran et al [10]. For the undirected MDD-friend network, we adopted an Erdös-Rényi model (resulting network was called Erdös-Rényi model-friend) and the Watts-Strogatz for simulation purposes [33]. The Watts-Strogatz model starts with a ring-shaped network, in which each node has seven neighbors, and set the rewire probability to 0.45.

## Results

### Results of the Linguistic Analysis

We collected the full information of the MDD group with 2,281,678 words written by 5013 group members in 3565 threads. There were a total of 5050 users in the MDD group, but 37 users deleted their Douban accounts making the content of their messages inaccessible; therefore, we have the information about their social networking behaviors without the actual text content of their posts. So we analyzed the linguistic patterns of 5013 users instead of 5050 users in this section. In all, 74.82% (1,707,151/2,281,678) of the words in our dataset were tagged into one or more categories defined by the SC-LIWC dictionary. Compared to the tag rate of other studies using LIWC/SC-LIWC [24], this tag rate is reasonably high and representative.

**Figure 2:**
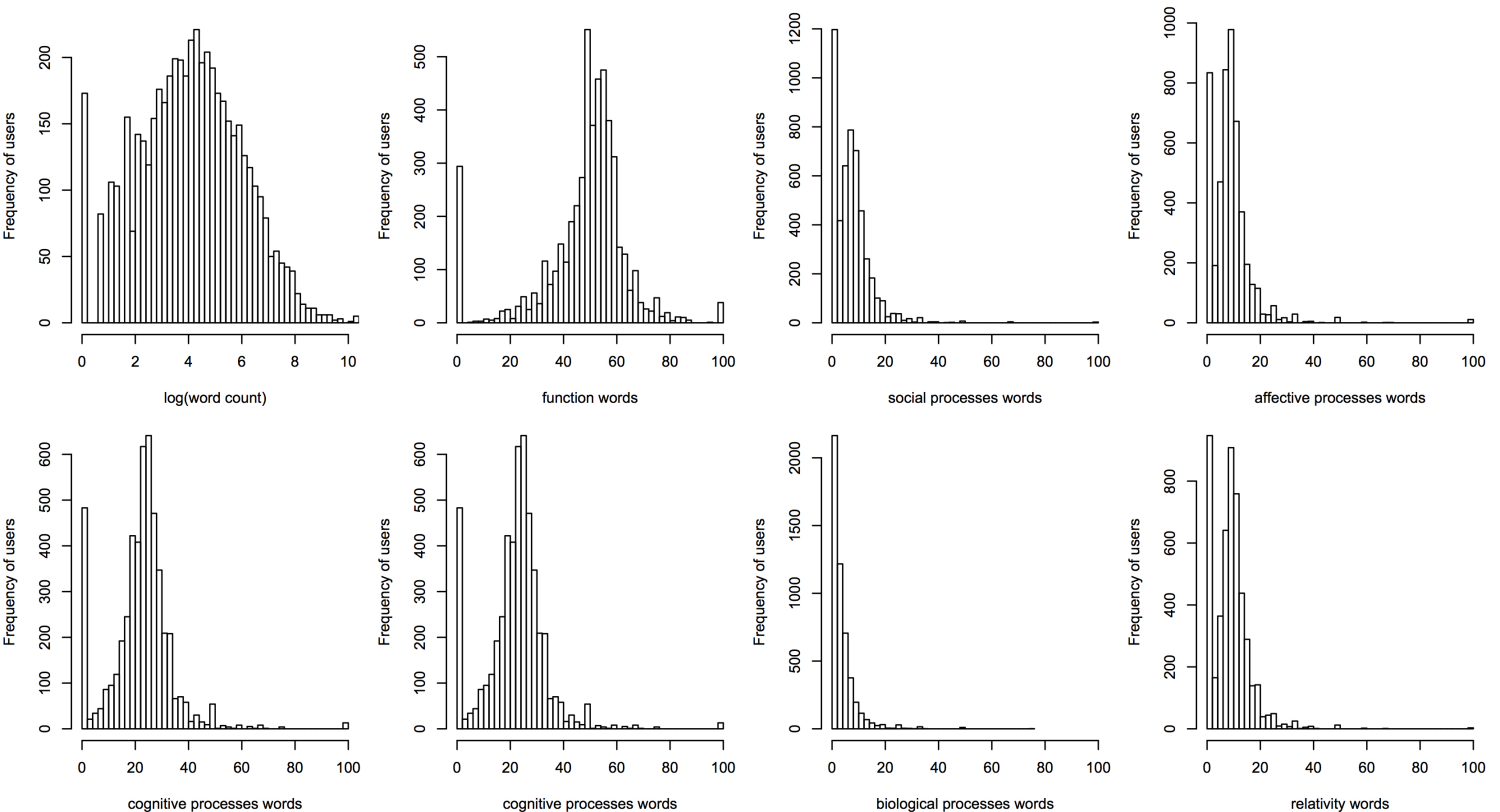
Distributions of word count and the seven main categories of words.

**Figure 3:**
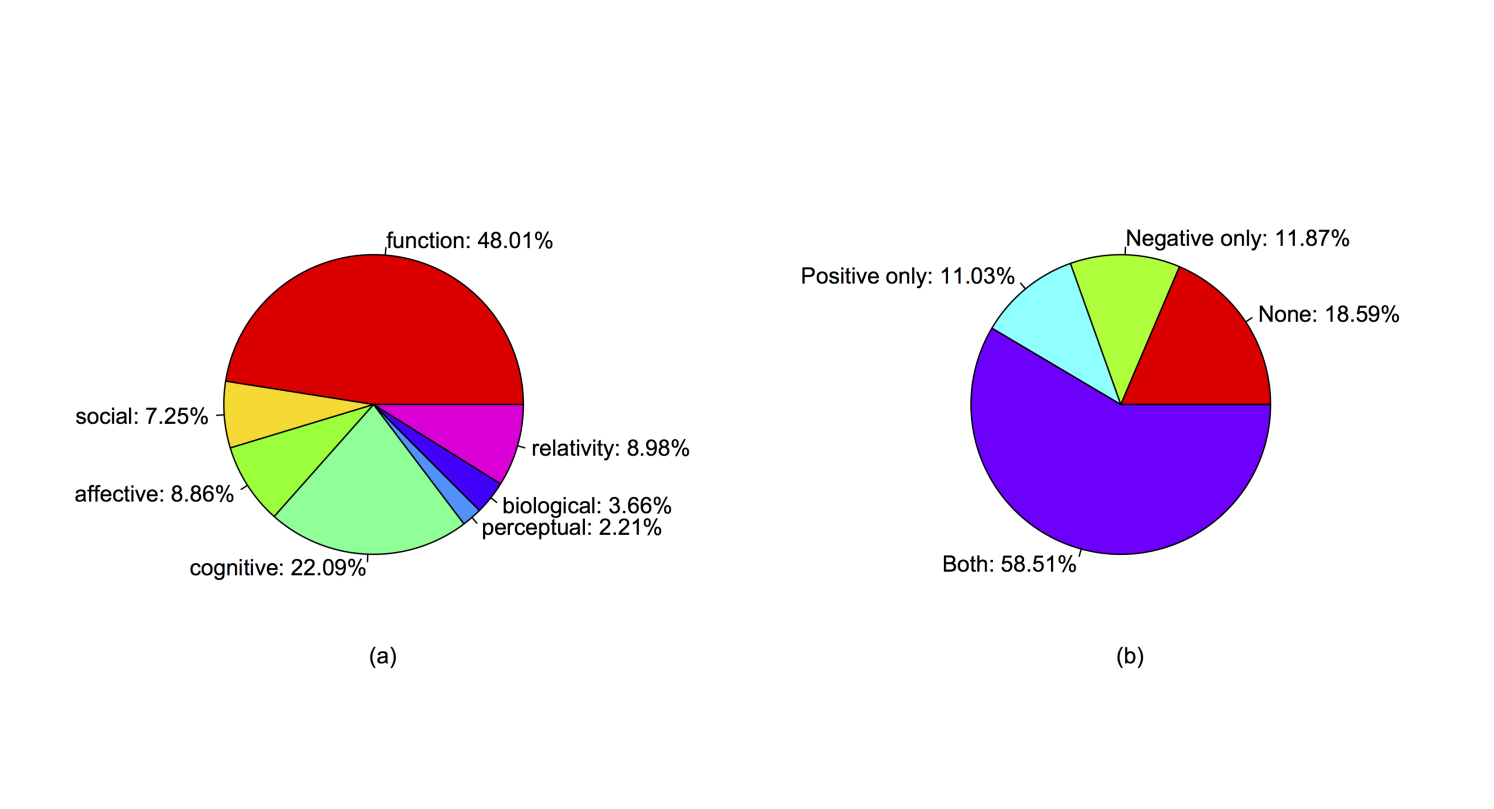
Pie charts of (a) the mean occurrence rates of the seven main word categories and (b) the proportions of members (N=5013) using negative and positive words.

The distributions of the word count and the seven main categories are shown in Figure 2. The occurrence of certain categories (eg, affective processes, cognitive processes) is 100% because of the existence of short messages containing only words from one category. The occurrence of function words and cognitive processes words peaked at approximately 50% and 20%, respectively. Other categories had relatively lower occurrence frequencies. The mean occurrence rates of the seven main categories are labeled in Figure 3a. On average, function words and cognitive processes categories accounted for the largest parts of the words used.

Previous psychological studies observed that style words made up approximately 55% of all the words people speak, hear, and read [25]. The style words include, but are not restricted to, function words and relativity words. In the MDD group, the style words (as represented by function words and relativity) accounted for nearly 60% of the whole corpus (summing the two occurrence rates in Figure 3). This finding is consistent with real-world psychological experiments indicating that the writing styles of depressed people (represented by the members of MDD group) are consistent both online and offline.

According to Beck’s cognitive theory of depression, self-preoccupation and negative thought content are the characteristics of depression [34]. This motivated us to verify whether the members in the MDD group also had these characteristics as revealed by the use of words related to self-preoccupation and negative thoughts. It is worth noting that although using the occurrence rates of positive and negative words gave us a straightforward view of the emotion polarization of the users, it was limited with little concern about the grammar and semantic features. Developing specific methods to quantify the sentiments and semantics is our future work.

**Figure 4:**
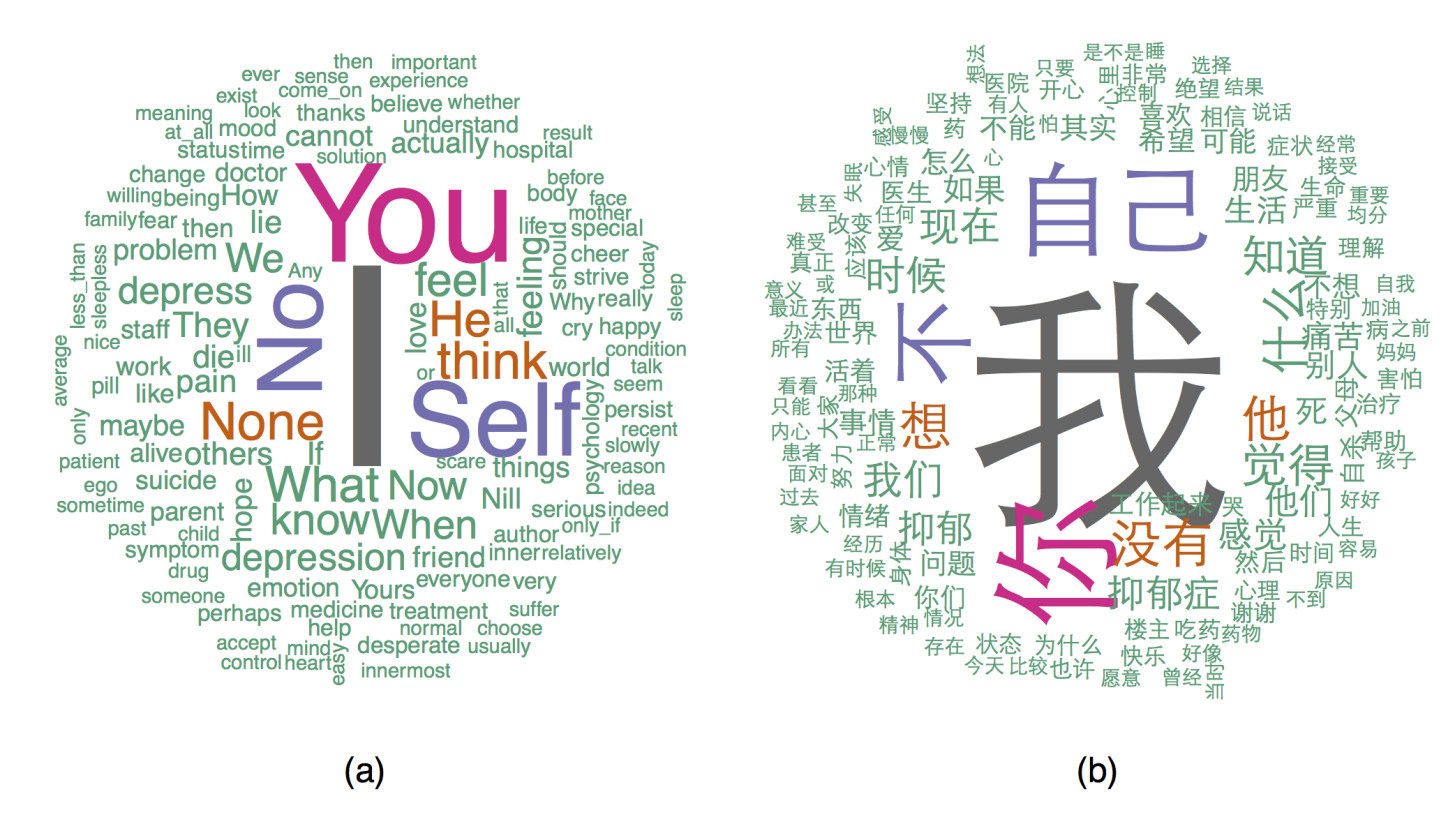
Word clouds of the content of the conversations in the MDD group in (a) translated English and (b) Chinese.

We first calculated the frequency of individual words and drew the word clouds for better visualization. Figure 4 shows both the Chinese and translated English versions. We observed the intensive use of self-focus words, such as “I (我)” and “self (自己),” and negative words, such as “no (不)” and “none (没有).” We then examined the subcategories under affective processes (positive and negative) and pronoun (first-person pronoun and second-person pronoun).

**Figure 5:**
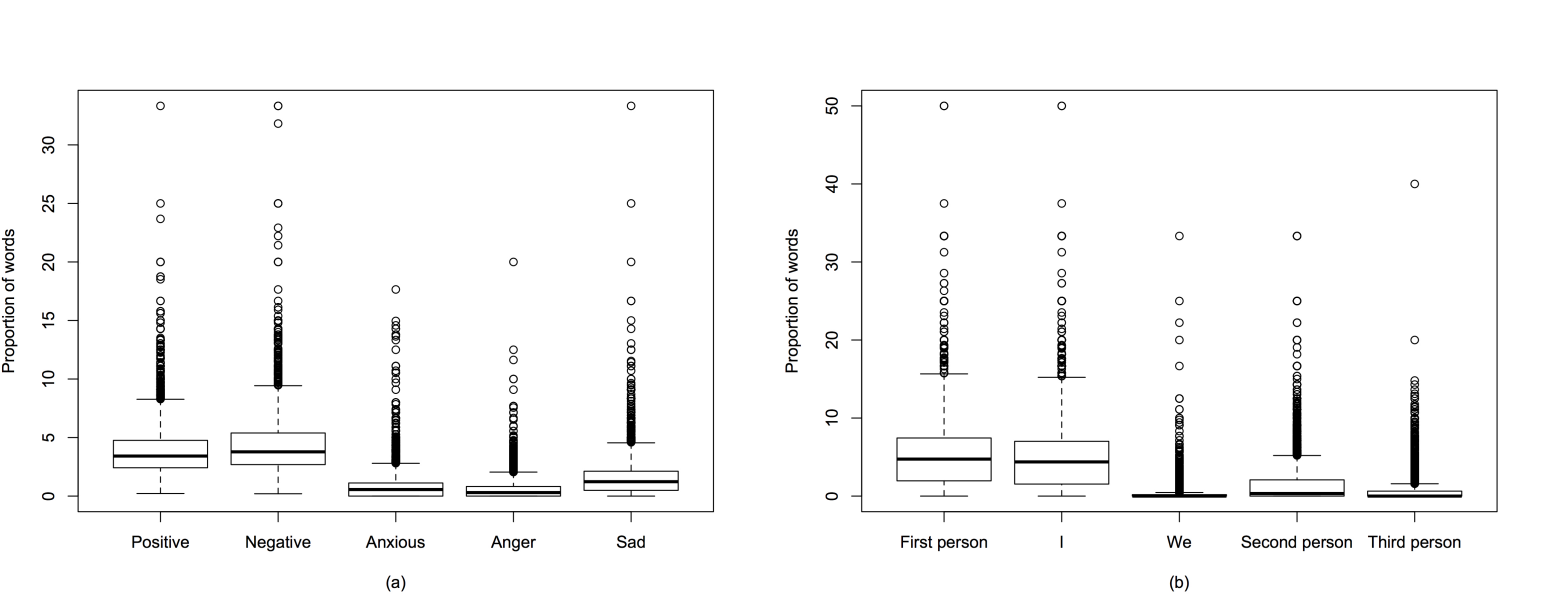
Box plot of the occurrence rate of two categorical words in the MDD group in the (a) affective processes and (b) pronoun categories. The horizontal line within each box represents the median. The top and bottom borders of the box are the 75th and 25th percentiles, respectively. The whiskers above and below the box mark the 90th and 10th percentiles. The points beyond the whiskers are outliers beyond the 90th percentile.

The affective processes category has negative and positive subcategories. Negative words can be further divided into anxious, angry, and sad words. In the MDD group, there were 11.87% (595/5013) of users who used negative words but not positive words; 11.03% (553/5013) of users used positive words but not negative words. In all, 58.51% (2933/5013) of users used both positive and negative words in their messages and 18.59% (932/5013) used neither positive nor negative words, as shown in Figure 3b. To gain a more detailed understanding, the box chart of the occurrence rate of affective processes words of the members who used both positive and negative words is shown in Figure 5a. The mean occurrence rate of the positive and negative words was 4.06 (SD 2.67) and 4.48 (SD 2.99), respectively (*t*_5499_=5.50, *P*<.001). Statistically, more negative words were used. This finding indicates that, in general, the users in the MDD group posted more negative content than positive.

The pronoun category had five subcategories, in which “I” and “we” were combined into first-person pronouns, “you” belonged to second-person pronouns, and “she,” “he,” and “they” belonged to third-person pronouns. The first-person pronouns-only or second-person pronouns-only members were not differentiated because both values were small (<5%). Similar to the affective processes category, we analyzed the occurrence rates of pronouns words for members who used all three types of pronouns and the results are shown in Figure 5b. Obviously, the first-person pronoun (especially the first-person singular pronoun) words were used more often than other pronouns. This may imply that a member in the MDD group wanted to talk about “their” versus “others” stuff. This finding is consistent with the psychological observations of depressed college students [34].

To summarize, more intensive use of negative and first-person pronoun words verified the characteristics of self-preoccupation and the negative content focus, which indicated that the users of the MDD group possessed the two characteristics of depression depicted by Beck’s cognitive theory. In addition, the MDD group members also revealed additional linguistic signals of depression (eg, the occurrence rates of the seven main categories), which could be further used in the surveillance and detection of depression in public health. These findings were also verified by aggregating the messages on individual threads (refer to Multimedia Appendix 1 for more details).

### Results of Network Analysis

**Figure 6:**
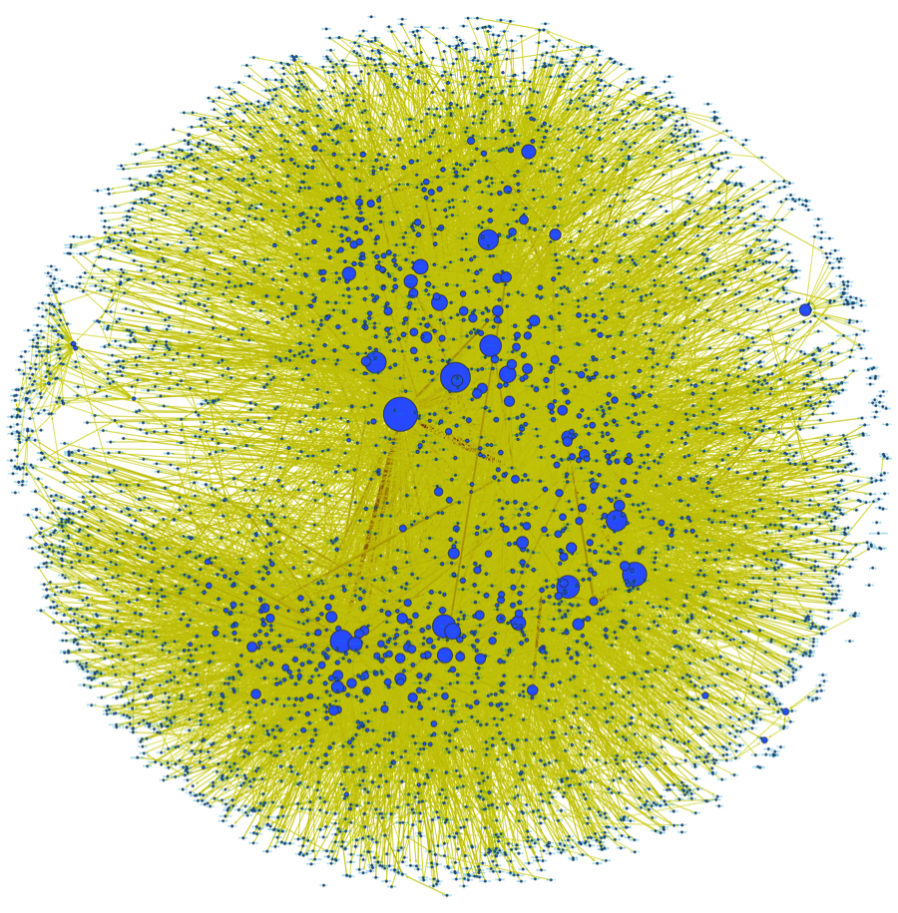
Visualization of the MDD network. The size of the node is proportional to the in-degree of the node and the darkness of the edge represents the edge betweenness centrality value.

In the MDD network, there were 5050 nodes (representing individual user IDs) and 36,657 edges (representing the reply-to relations of the messages posted by two corresponding user IDs). It is worth noting that although 37 members deleted their account, we still had access to the information on their conversations with others except for the content of their messages. We excluded self-loops; the network is visualized in Figure 6. This network had 162 weakly connected components. The largest one consisted of 96.69% (4883/5050) of all nodes, indicating that 96.7% of the nodes could reach one another through an undirected path in the same giant component. According to the definition of the edge, 3565 self-loops stood for the threads initialized by group members and the other 135 ones stood for the messages that the initiators left in the thread that was created but did not specify the targeted members. The self-loops would be removed in later analysis of network structure properties because we focused on the conversations among MDD group members.

**Figure 7:**
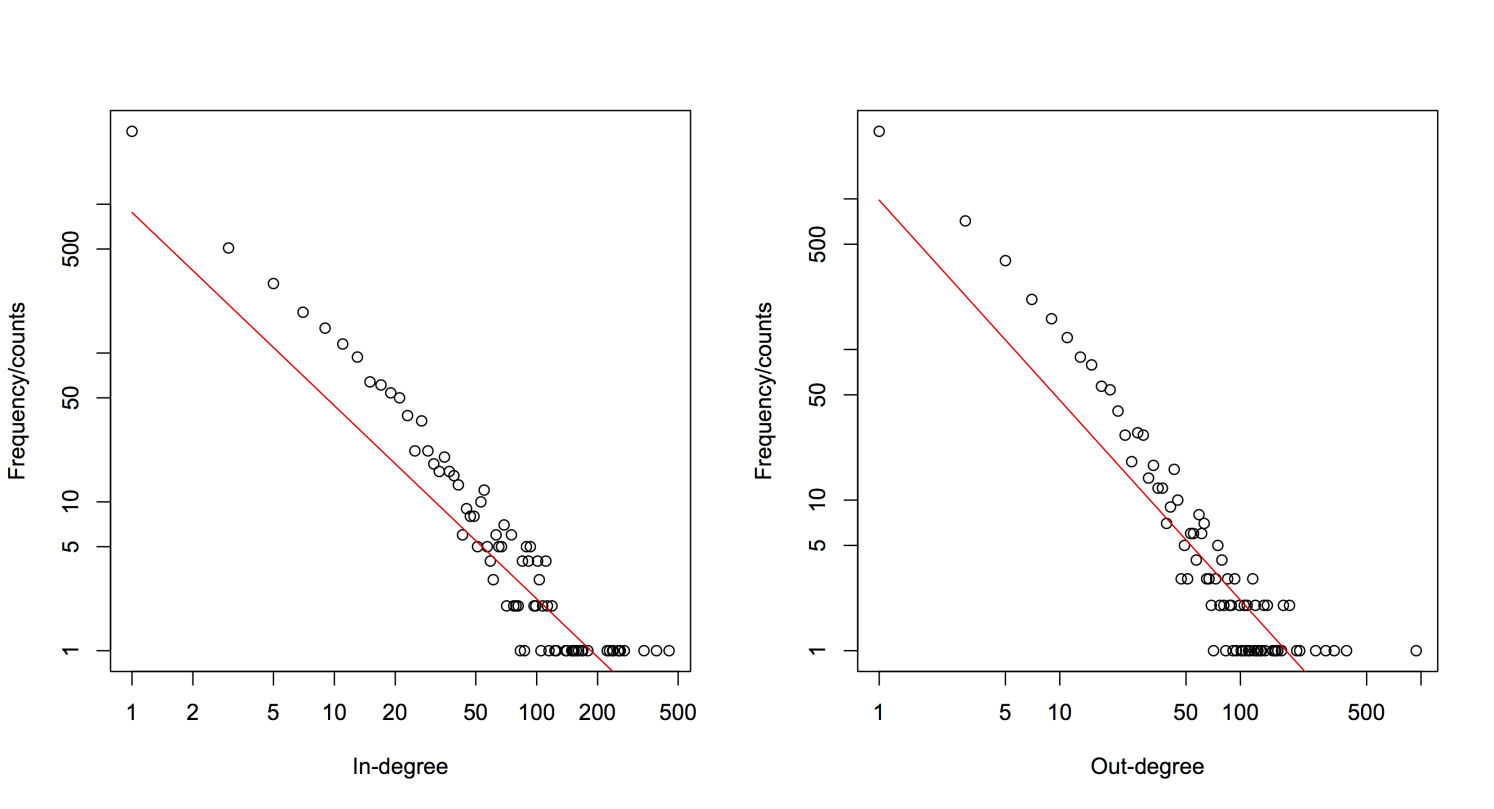
In-degree and out-degree distributions of the MDD network.

A node has both in-degree and out-degree. The distributions of in-degree and out-degree are shown in Figure 7. We found that both the in- and out-degree followed the power law distribution, with exponents of 2.13 and 2.20, respectively (shown in Table 1). The difference of the exponents of the in-degree and out-degree revealed the imbalanced tendency of user behaviors in posting messages (self-expression), posting replies (active peer communication), and receiving replies from others (peer response).

**Table 1:** Comparison of the MDD network, Myspace (in [35]), MedHelp friendship networks (in [10]), and other simulated networks.

Network metric	MDD	Myspace	Erdös-Rényi model	Barabási-Albert model	MDD-friend	MedHelp	Erdös-Rényi -friend model	Watts-Strogatz model
Type	Directed	Directed	Directed	Directed	Undirected	Undirected	Undirected	Undirected
Nodes	5050	36,459	5050	5050	5050	30,915	5050	5050
Edges	36,657	80,675	36,657	36,657	17,401	113,273	17,401	17,401
Connected components	162	1	1	1	162	2	5	2
Nodes in the largest weakly connected component	4881	36,459	5050	5050	4881	30,870	5046	5049
Network density (10^–5^ scale)	143.8	6.07	143.8	138.6	136.5	23.7	136.5	136.5
Network diameter	10	11	8	11	11	—	8	8
Network reciprocity (10^–2^ scale)	34.0	1.45	0.08	0.03	—	—	—	—
Clustering coefficient (10^–2^ scale)	4.47	0.031	0.29	0.64	4.47	3.1	0.13	1.19
Average shortest path length	4.11	5.14	4.53	2.83	3.80	3.81	4.64	4.69
Power law exponent (in/out)	2.13/2.20	2.65/1.99	—	2.15/3.01	2.29	2.12	—	—
Min degree (in/out)	0/0	1/0	5/5	0/4	0	—	0	3
Max degree (in/out)	451/942	558/6077	19/20	6053/7	464	—	19	27

In the MDD network, 36.03% (1820/5050) of nodes had zero in-degree, indicating that there was no other member who replied to their messages in the MDD group. On the other hand, the in-degree could go as large as 541. For the out-degree, 50% of members had a value of either 1 or 2, whereas the largest was 991. We examined the node with the largest in-degree and the node with the largest out-degree. It turned out that it was the same node that had both the largest in-degree and the largest out-degree, indicating that this user was the most active member in the group from both perspectives. This member was also one of the administrators invited by the group initiator. This finding indicates that additional rewarding and ranking mechanisms (eg, coadministrators or facilitators) would be useful to improve the communications in the design of online health groups. In addition, the users with high in-degrees also had high out-degrees, indicating that the users had mixed behaviors. This will be further examined in the next section.

To better understand the unique features of the conversations in the MDD group, we compared the topological properties of the MDD network with the conversation-based network of Myspace [35], the friendship-based MedHelp network [10], and model-generated networks. The results are shown in Table 1. Comparatively, the MDD network and MDD-friend were much denser and more clustered than Myspace and MedHelp as indicated by the large values for network density and clustering coefficient. This indicates that the local neighborhood of a member in MDD tended to have more connections than others. With a small average shortest path length and a large clustering coefficient, the MDD network possessed the small-world property as compared with Erdös-Rényi random graph model.

In addition, the reciprocity of the MDD network was approximately 30 times higher than that in Myspace. That means if a member *A* replied to a message posted by another member *B* in the MDD group, the chance that *B* would also reply to a message posted by *A* was 34%, whereas the chance was 1.45% in Myspace. In addition, 52% of node pairs in the MDD network had at least one directed path from one to the other, whereas the ratio was only 1% in Myspace. These results show that the MDD group was a very sticky community. The majority (more than 50%) of users could be reached by directed paths. This finding is verified by examining the Bow-Tie model in Table 2.

**Table 2:** Comparison of Bow-Tie model analysis between the MDD network and other networks.

Networks	MDD	Myspace	Java forum	Web	Erdös-Rényi model	Barabási-Albert model
SCC	54.53%	1.17%	12.30%	27.70%	99.91%	0.10%
IN	29.27%	0%	54.90%	21.20%	0.04%	98.10%
OUT	7.80%	81.50%	13.00%	21.20%	0.04%	0%
TENDRILS	4.22%	0.027%	17.50%	21.50%	0%	0.61%
TUBES	0.04%	0%	0.40%	0.40%	0%	0%
DISC	4.22%	17.30%	1.90%	8.00%	0.01%	1.21%

The Bow-Tie model was used to examine the general structure of the network and its reciprocity in more detail. As shown in Table 2, the MDD network was very different from both the networks of Java forum and the Web. Compared with other networks, the MDD network had a much bigger SCC (more than 50%). This indicates that more than 50% of the members in the MDD group could mutually reach one another through a directed path within the SCC. The fraction of SCC in the MDD network was much higher than the fractions of SCC in Myspace (1.17%), Java forum (12.30%), and the Web (27.70%). This finding, together with the high reciprocity and high clustering coefficient values described previously, indicates that the members of MDD formed a sticky social group within which the spread of information was very efficient. These findings generate critical insights about the network structures that facilitate the information diffusion in a social group. Applying the insights to public health research and practice, it can help health providers identify better strategies to promote health-related information in social media and control the spread of negative information.

Note that the definition of the edge direction in the Java forum was the opposite of ours. Therefore, the IN (OUT) component in the Java forum should be OUT (IN) by our definition. Members belonging to the in component behave like members who only answer questions in the Java forum and these members posted messages to others but never got any response. Both the Java forum and the MDD network had a large in component (29.27%) and the proportion of in component was almost four times larger than the out component (7.80%). This also revealed the imbalanced tendency that members in the MDD group would have liked to express their points without getting any responses. To alleviate the heterogeneous behaviors of in and out, some special encouraging functions could be prompted to the users with imbalanced communications when designing/organizing health forums to improve online communications.

### Relations Between Linguistic and Topological Properties

The position of a member in a social network represented his or her role in this social group. The roles of members were potentially associated with the language use of the members. A key question that remained unanswered was “What is the relationship between the language use and the topological positions in the network?”

**Figure 8:**
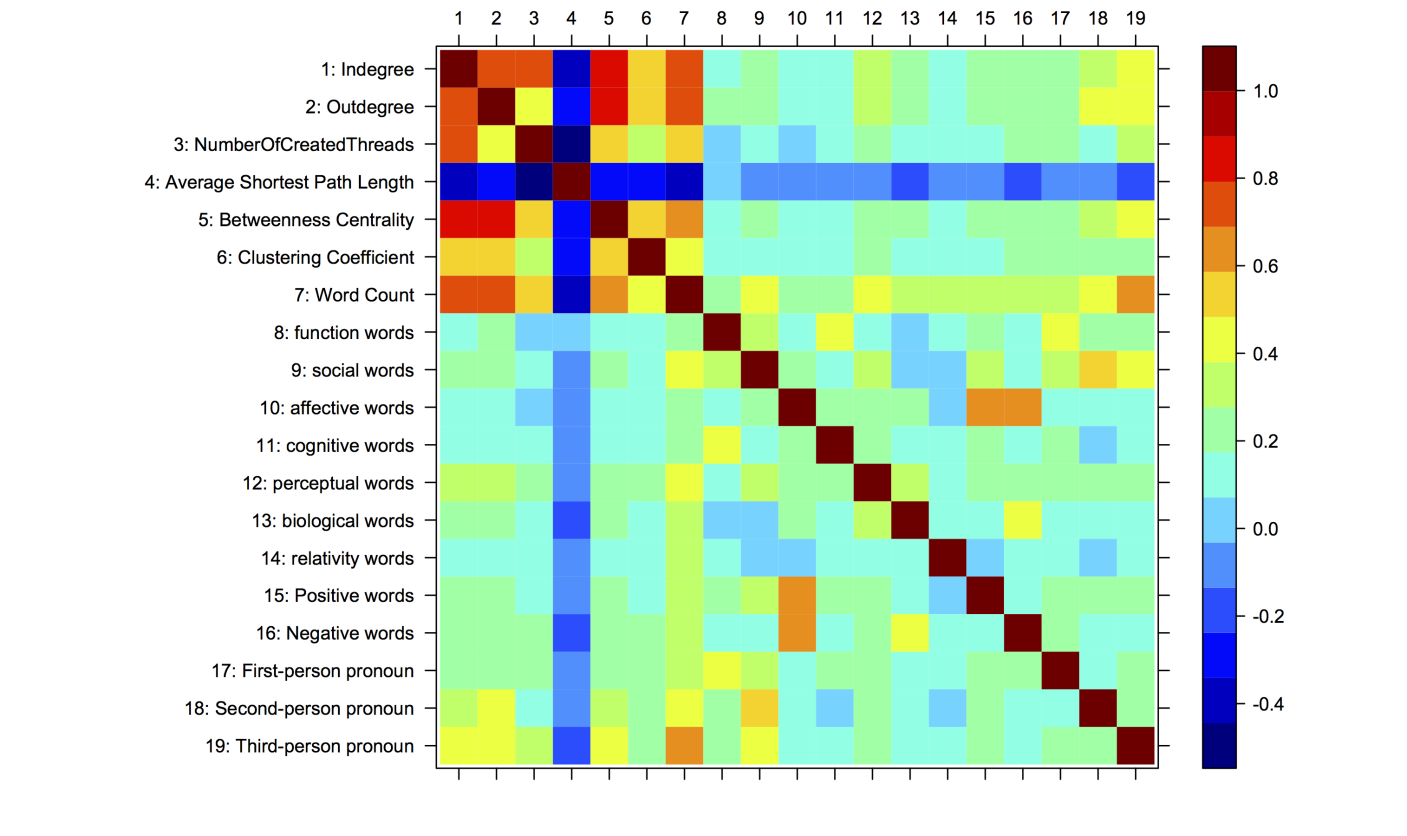
Spearman correlations of the topological (1-6) and linguistic (7-19) properties.

To answer this question, we integrated the linguistic properties and the topological properties observed from the previous two sections. We chose two sets of representative linguistic and topological properties: (1) the word count, the seven categories, and positive, negative, and pronoun words (first-, second-, and third-person); and (2) degree, including in-degree and out-degree, the number of self-loops representing the initiated threads, average shortest path length, betweenness centrality, and clustering coefficient. Spearman correlations were run to assess the relationship between the two sets of properties using the 5013 members in the MDD group; the results are shown in Figure 8.

In terms of the relations between topological and linguistic properties of the same sample size, we found that the correlations were moderately strong (coefficient >.3 or <-.3 [36]) for word count, second-person pronouns, and third-person pronouns, as shown in the left-lower part in Figure 8. The word count had a mild negative association (ρ=-.36) with average shortest path length and moderate positive correlations (ρ>.45) with other topological properties. These findings indicate that, in general, the length of the messages users posted were associated with their topological position/locations in the social network. In addition, there were also moderate positive correlations between both second-person pronouns and third-person pronouns with the in-degree (ρ=.32 and ρ=.42, respectively) and the out-degree (ρ=.43 and ρ=.42). This implies that when a user used the second- or third-person pronouns more intensively, this user had a higher chance of also possessing a higher in-degree and out-degree. The length of the content was still one of the dominating factors, potentially because the longer messages have a higher chance to consist of these linguistic factors.

There was a strong correlation (ρ=.70) between the in-degree and the out-degree of a member. This indicated that if a member posted more messages to other members, this user had a higher chance of receiving more replies from others and vice versa. In addition, there was also a strong correlation between the number of threads a user created and the in-degree and out-degree of the member (ρ=.74 and ρ=.40, respectively), which was expected from the definition of the MDD network.

**Figure 9:**
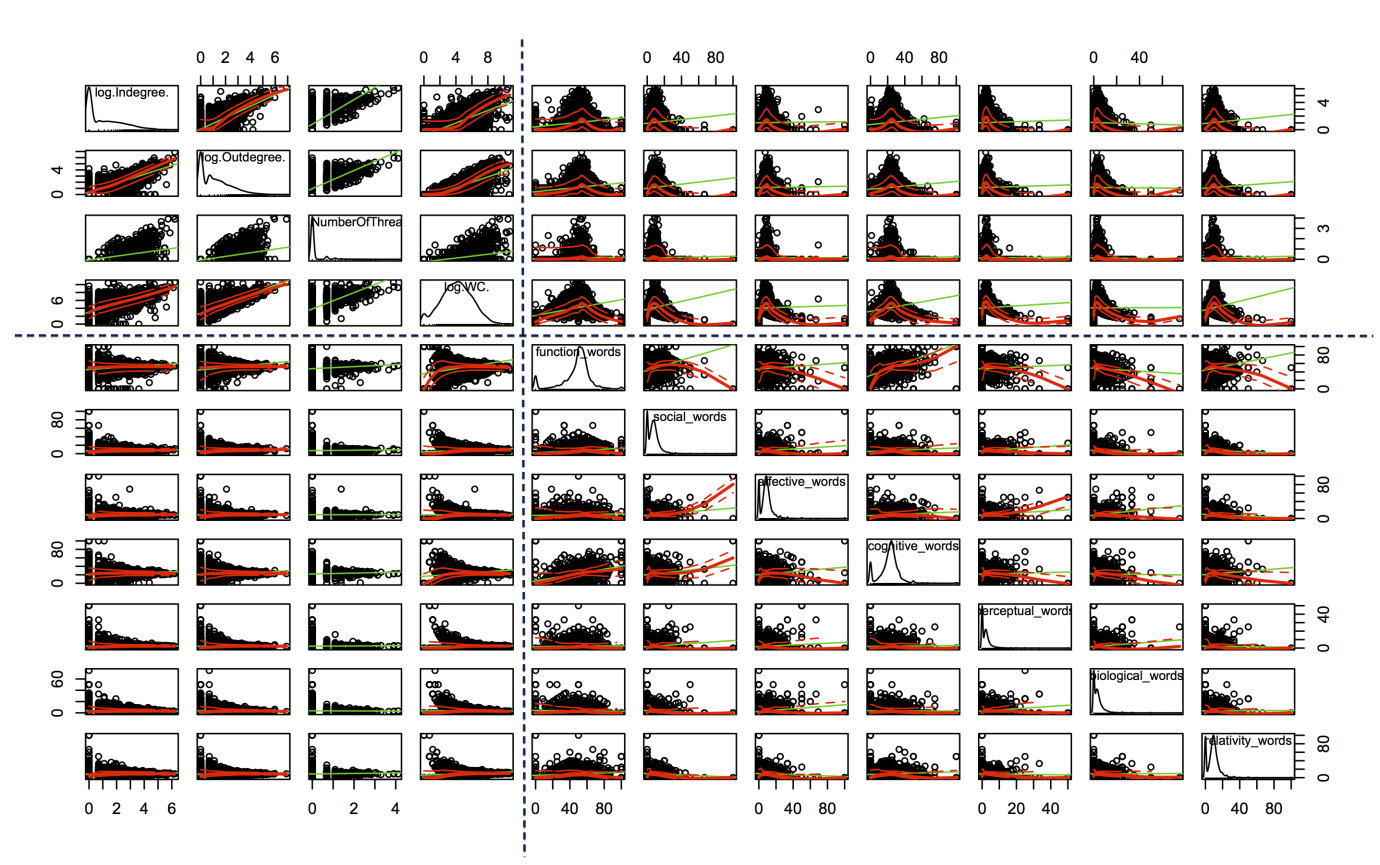
Scatterplot of the relationship between topological (in-degree, out-degree, the number of threads [NumOfThreads]) and linguistic properties (word count [WC] and 7 main categories) (part 1).

**Figure 10:**
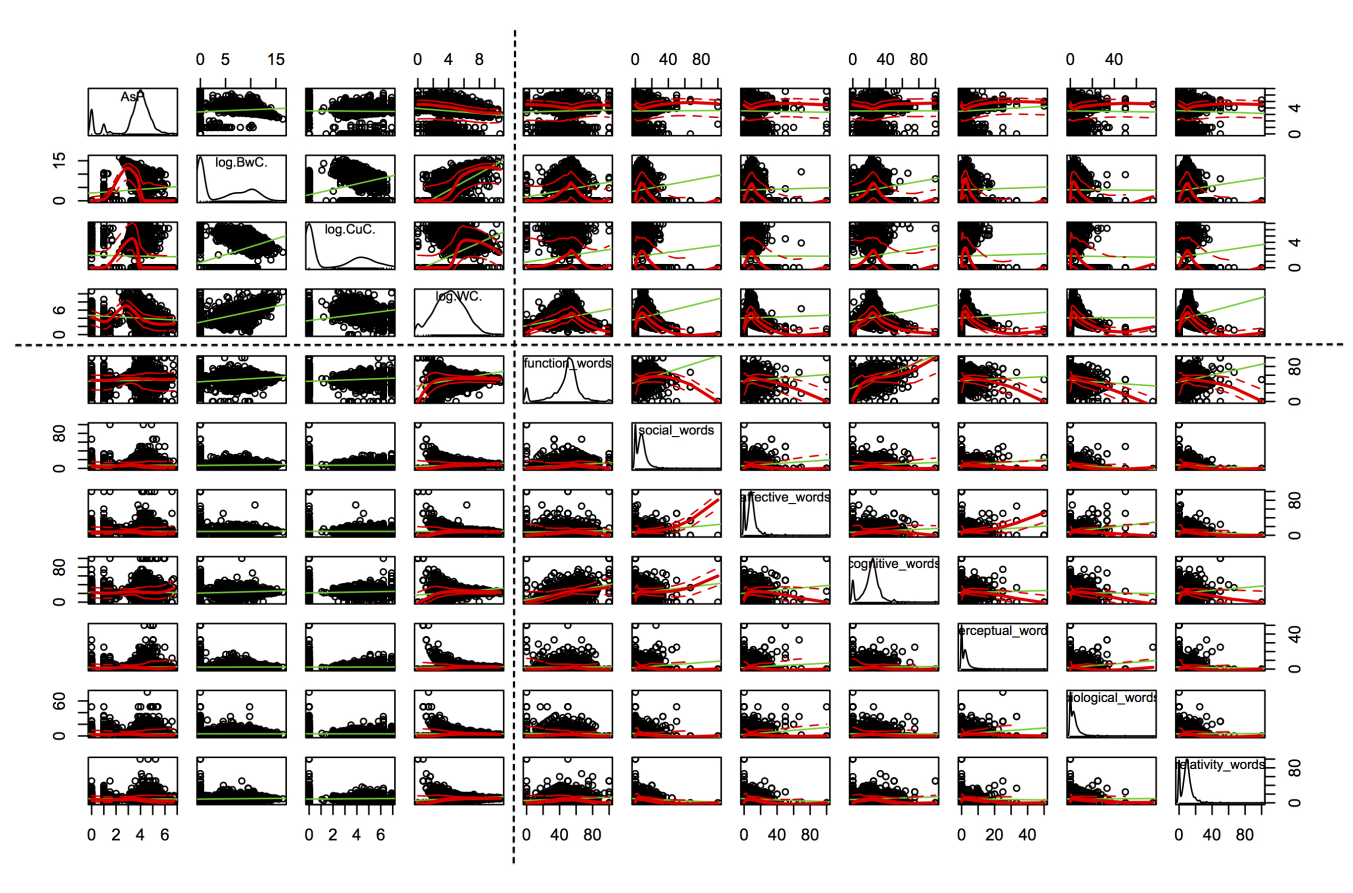
Scatterplot of the relationship between topological (average shortest path length [AsP], betweenness centrality [BwC], clustering coefficient [CuC]) and linguistic properties (word count [WC] and 7 main categories) (part 2).

**Figure 11:**
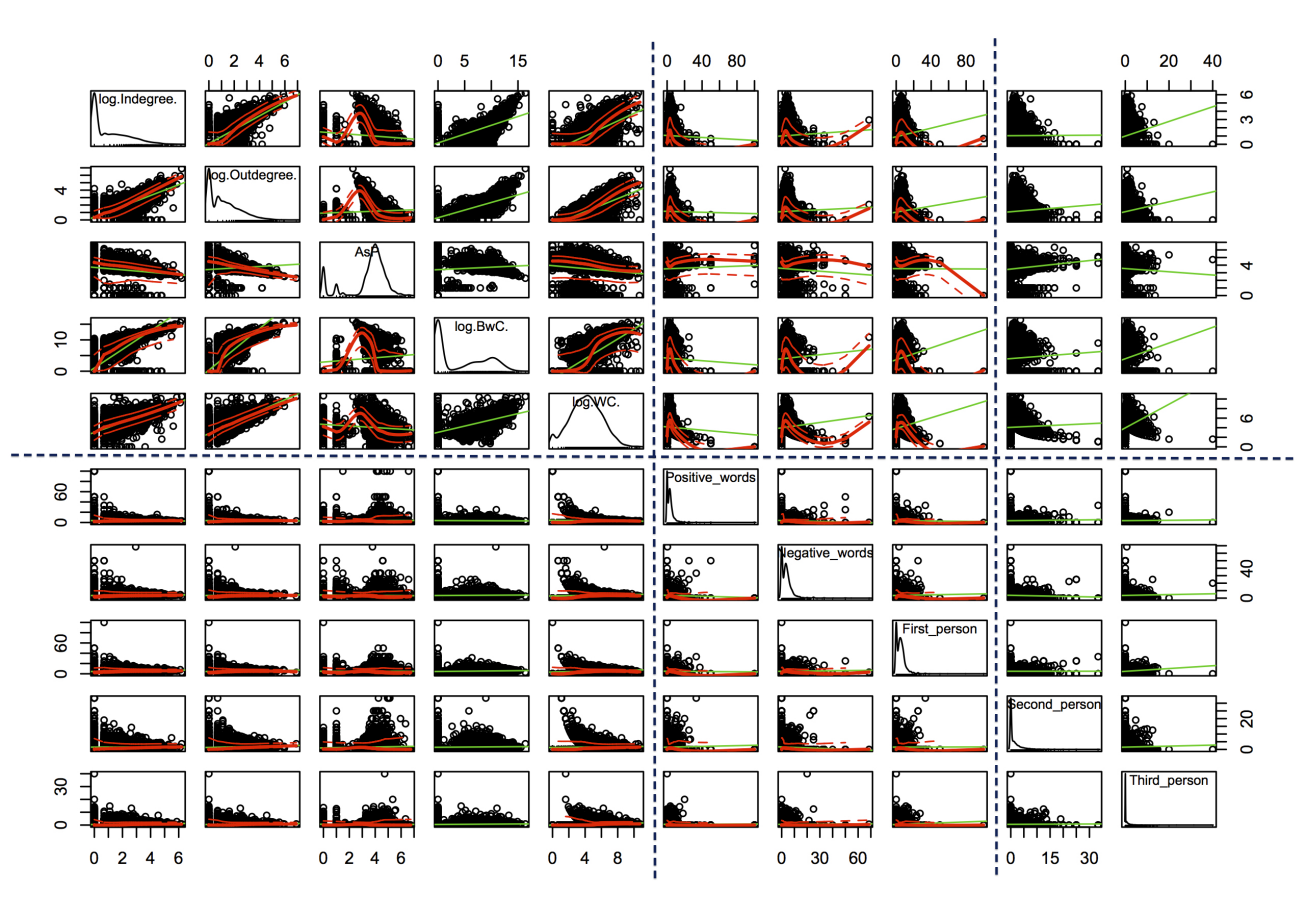
Scatterplot of the relationship between topological (in-degree, out-degree, average shortest path length [AsP], betweenness centrality [BwC]) and linguistic properties (word count [WC], detailed positive and negative affective and pronoun words) (part 3).

Figures 9-11 present the scatterplots of the relationships between 19 topological and linguistic properties. Each row and column denotes a particular property. The distributions of individual properties are shown in the diagonals of the scatterplot matrices. The red curve in each subfigure denotes the mean values of the vertical property over the horizontal property. The green curve denotes the best-fitted linear regression of the plots. Note that the distributions of in-degree, out-degree, the number of threads a user created, betweenness centrality, clustering coefficient, and word count followed power law distributions, so we used the log function of these properties to scale down. Average shortest path length and the occurrence rates of categorical words followed multimodal distributions with two or more local peaks. The dotted lines separate the topological and linguistic properties in the scatterplot matrices.

We observed monotonic relations between a set of linguistic properties (word count, second-person pronouns, third-person pronouns) and the topological properties, and also between the topological properties (in-degree, out-degree, the number of threads a user created, average shortest path length, betweenness centrality, and clustering coefficient). Refer to the upper-left parts in Figures 9-11 and the upper-right part in Figure 11. These monotonic relations explained the strong correlations between word count, second-person pronouns, third-person pronouns, and the topological properties (observed in Figure 8).

Conversely, we did not find clear monotonic relations between other pairs of linguistic (including the seven main categories*,* positive emotion words, negative emotion words, first-person pronouns) and topological properties (except average shortest path length). However, we observed interesting convergent relations. Refer to the upper-right parts in Figures 9 and 10 and the upper-middle part in Figure 11. In general, the nonmonotonic curves could be divided into two phases: monotonically increase (phase 1) and decrease (phase 2). These curves explained the weak correlations between these properties because the Spearman correlation was only feasible to capture the monotonic relations. The convergent patterns indicated that when the values of the topological properties increased, the linguistic properties (the seven main categories*,* positive emotion words, negative emotion words, first-person pronouns) tended to converge to the mean gradually. These convergent and two-phase relations indicated the self-organizing behaviors of users in the MDD group. The factors lead to these convergent patterns remaining hidden and they will be examined in detail in our future work.

### Discussion

In this paper, we characterize both the language use and the network properties of a popular online health group for MDD in China. For language use, we aggregate messages on members and verify the characteristics of self-preoccupation and negative focus of depressed individuals revealed in previous psychological studies and in other social media platforms. For network properties, the MDD network differentiates from other social networks with a highly sticky structure, imbalanced in-degree and out-degree, and a high reciprocity. By integrating these two types of properties, we find a set of interesting correlations and interesting convergent relations between the linguistic and the topological properties.

This work sheds light on the in-depth understanding of how Web users communicate with one another in MDD online health groups. The analysis of language use helps understand the expression of depression on a large scale. The results provide important insights for depression surveillance in public health. Our findings help explain the dissemination of depression-related information in a highly mutually connected community devoted to depression (the MDD group). The social network analysis presents novel and efficient information spread patterns of the MDD group that can be further adapted by health care providers to develop better and effective functions to facilitate online communications in the design of Health 2.0 applications.

There are also a number of limitations and questions that need further investigation:

1. How to identify the topics of the discussions in MDD group and other online health communities? We plan to adapt state-of-the-art text-mining methods into the linguistic analysis with LIWC in our future work to address this issue.
2. How to propose new network models to describe and replicate the unique topological properties of the MDD group? We are now developing a new generative network model based on the basic Barabási-Albert model with a focus on being able to control the value of the reciprocity of the network to replicate the higher intendancy of mutual communications in the MDD group. We will also develop models of multiple (interdependent) networks [19,21].
3. Do these unique topological properties only exist in the MDD group or in other online health groups as well? We are collecting data from online health groups for different mental health problems and other types of diseases on different social media platforms. Empirical studies with the new data will be our future work.

## Appendix

Multimedia Appendix 1 This supplementary file contains content, figures, and tables that support the conclusion of the paper, but are too redundant to be included into the main manuscript. There are two figures in this document (in the text). The high resolution versions of the supplementary figures were uploaded to the system as well.

## Abbreviations

LIWC: Linguistic Inquiry Word Count
MDD: major depressive disorder
SC-LIWC: Simplified Chinese LIWC
