# Supplementary material for: Understanding Online Health Groups for Depression: Social Network and Linguistic Perspectives
Source: J Med Internet Res. 2016 Mar 10;18(3):e63. doi: 10.2196/jmir.5042 (PMC4807247; doi:10.2196/jmir.5042)
Supplement: Multimedia Appendix 1 [file jmir_v18i3e63_app1.pdf]

Supplementary Materials

Ronghua Xu, Qingpeng Zhang (qingpeng.zhang@cityu.edu.hk)
Understanding Online Health Groups for Depression: Social Network and Linguistic Perspectives
J Med Internet Res 0000;##(##):e##
URL: http://www.jmir.org/0000/0/e0/
doi:10.2196/jmir.5042

Word Use of Individual Threads

After a discussion thread was created in the MDD group, members could join it by posting messages under the corresponding thread. There are various reasons that a member replied to a thread — either being interested in the topic or sharing similar experience of the topic. It is intuitive to assume that the MDD group members share similar language use patterns within the same corresponding thread because of the similarity of their interests and experience. In this part, we analyze the language use of individual threads by aggregating all the messages belonging to the same thread and compare the it with the results of individual users.

Similar to the frequency of users, the mean occurrence rates of the seven main categories in individual threads are labeled in Supplementary Figure 1(a). On average, *funct* and *cogmech* categories represent the largest parts of the words being used in a thread. And a slightly different *relativ* (mean 9.29%, and standard deviation, *SD*=4.41) and *affect* (mean 9.22%, SD=4.94) words (t value = 0.72, $P < .5$) are used.

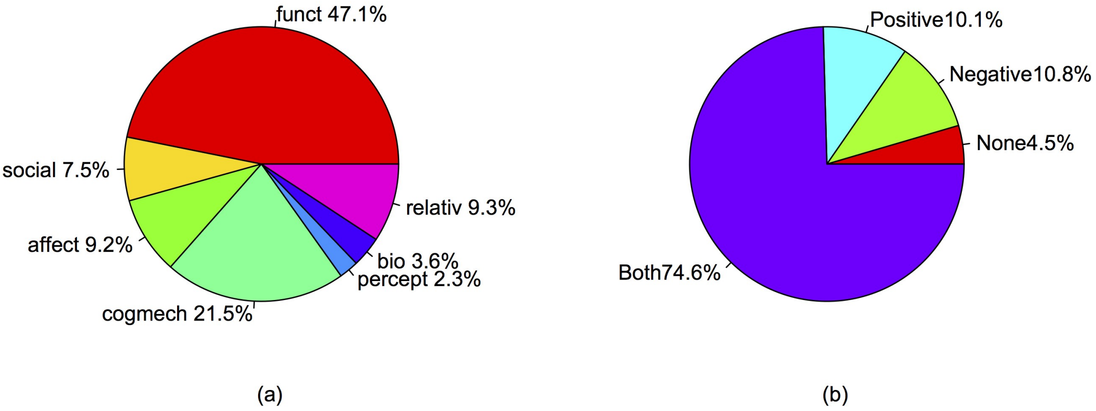

Supplementary Figure 1. The pie charts of: (a) the mean occurrence rates of the seven main word categories, (b) the proportions of discussion threads using both negative and positive words.

In terms of *affect* words, 11.7% of threads only had negative words, and about the same number of threads had only positive words, as shown in Supplementary Figure 1 (b). For threads with both negative and positive words, the word use distributions are shown in Supplementary Figure 2. Similar to the word use patterns of the individual users, the difference of threads between negative and positive words is significant with a mean of 4.04 (*SD* = 2.32) for negative words, and 3.74 (*SD* = 2.37) for positive words (*t*-value = 4.70, *P* < .001). In terms of *pronoun* words, the first person pronouns were more frequently used than

the other two kinds of pronouns, as shown in Supplementary Figure 2 (b). The patterns are the same as that observed in individual members.

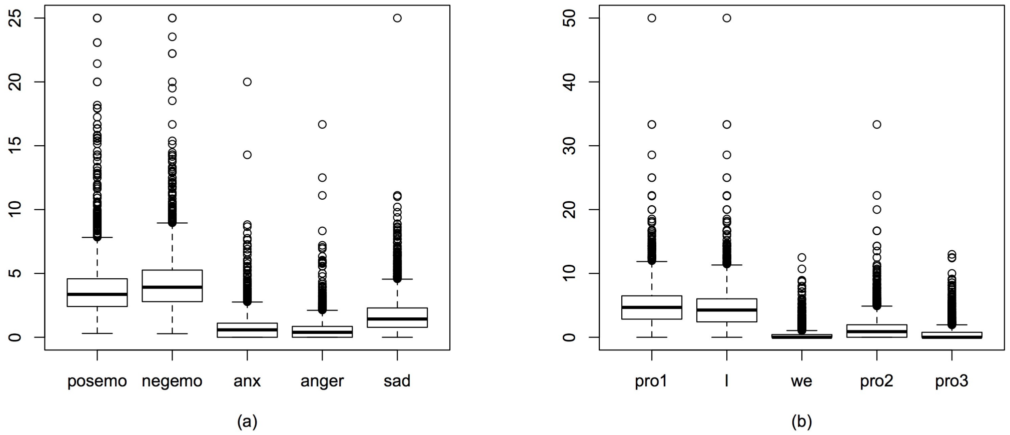

Supplementary Figure 2. Box plot of the occurrence rates of two categorical words in the threads, (a) *affect* categories, (b) *pronoun* categories.

Extended Table 1

In this extended table, we added one more interest group from *Douban*, named *Scientist* (http://www.douban.com/group/scientists/). The reciprocity of MDD is almost seven times higher than that of Scientist group, with 34% and 4.8% respectively. The results showed that the property of high mutual communication is not inherited from the *Douban* platform and verified the unique stickiness of the MDD group. More comparison results of other groups can be found in the paper below,

‘Xu, R. & Zhang, Q. (Nov 2015). *Social Dynamics of the Online Health Communities for Mental Health*. International Conference of Smart Health. Phoenix. USA. Nov 17-18, 2015.’

Supplemental Table 1. Comparison of the topological properties between the MDD group and the Scientist group.

Network metric	MDD	Scientist
Type	*dir*	*dir*
Node	5,050	1,691
Edge	36,657	4,421
Comp	162	70
WCC	4,881	1,611
Dens	143.8	154.7
Diam	10	13
Reci	34.0	4.8
CuC	4.47	3.0
AsP	4.11	5.23
Expo	2.13/<br>2.20	2.17/<br>2.83
Min deg	0/0	0/0
Max deg	451/<br>942	189/<br>198

In this table, *dir* is short for directed, and *Un-dir* for undirected; *Comp* denotes the number of connected components; *WCC* denotes the number of nodes in the largest weakly connected component; *Dens* is short for network density in 10^{-5} scale; *Diam* is network diameter; *Reci* is the network reciprocity in 10^{-2} scale; *CuC* is the clustering coefficient in10^{-2} scale; *Expo* denotes the in/out power law exponent; *Min deg* and *Max deg* denote the minimum and maximum in/out degree separately.
